# Evaluation of macular thickness and volume tested by optical coherence tomography as biomarkers for Alzheimer’s disease in a memory clinic

**DOI:** 10.1038/s41598-020-58399-4

**Published:** 2020-01-31

**Authors:** Domingo Sánchez, Miguel Castilla-Marti, Marta Marquié, Sergi Valero, Sonia Moreno-Grau, Octavio Rodríguez-Gómez, Albert Piferrer, Gabriel Martínez, Joan Martínez, Itziar De Rojas, Isabel Hernández, Carla Abdelnour, Maitée Rosende-Roca, Liliana Vargas, Ana Mauleón, Silvia Gil, Montserrat Alegret, Gemma Ortega, Ana Espinosa, Alba Pérez-Cordón, Ángela Sanabria, Natalia Roberto, Andreea Ciudin, Rafael Simó, Cristina Hernández, Lluís Tárraga, Mercè Boada, Agustín Ruiz

**Affiliations:** 1 Research Center and Memory Clinic, Fundació ACE, Institut Català de Neurociències Aplicades, Universitat Internacional de Catalunya, Barcelona, Spain; 2 Centro de Investigación Biomédica en Red de Enfermedades Neurodegenerativas (CIBERNED), Instituto de Salud Carlos III, Madrid, Spain; 3 Clínica Oftalmológica Dr. Castilla, Barcelona, Spain; 4 Department of Ophthalmology, Hospital de l’Esperança, Parc de Salut Mar, Barcelona, Spain; 5 Topcon España Clinical Affairs, Sant Just Desvern, Spain; 6 Faculty of Medicine and Dentistry, Universidad de Antofagasta, Antofagasta, Chile; 7 Iberoamerican Cochrane Centre, Barcelona, Spain; 8 Diabetes and Metabolism Research Unit and Centro de Investigación Biomédica en Red de Diabetes y Enfermedades Metabólica Asociada (CIBERDEM), Vall d’Hebron Research Institute, Barcelona, Spain

**Keywords:** Alzheimer's disease, Alzheimer's disease, Alzheimer's disease, Alzheimer's disease

## Abstract

Building on previous studies that report thinning of the macula in Alzheimer’s disease (AD) and mild cognitive impairment (MCI) patients, the use of optical coherence tomography (OCT) has been proposed as a potential biomarker for AD. However, other studies contradict these results. A total of 930 participants (414 cognitively healthy people, 192 with probable amnestic MCI, and 324 probable AD patients) from a memory clinic were consecutively included in this study and underwent a spectral domain OCT scan (Maestro, Topcon) to assess total macular volume and thickness. Macular width measurements were also taken in several subregions (central, inner, and outer rings) and in layers such as the retinal nerve fiber (RNFL) and ganglion cell (CGL). The study employed a design of high ecological validity, with adjustment by age, education, sex, and OCT image quality. AD, MCI, and control groups did not significantly vary with regard to volume and retinal thickness in different layers. When these groups were compared, multivariate-adjusted analysis disclosed no significant differences in total (p = 0.564), CGL (p = 0.267), RNFL (p = 0.574), and macular thickness and volume (p = 0.380). The only macular regions showing significant differences were the superior (p = 0.040) and nasal (p = 0.040) sectors of the inner macular ring. However, adjustment for multiple comparisons nullified this significance. These results are not supporting existing claims for the usefulness of macular thickness as a biomarker of cognitive impairment in a memory unit. OCT biomarkers for AD should be subject to further longitudinal testing.

## Introduction

The diagnosis of Alzheimer’s disease (AD), the most frequent neurodegenerative disease, requires clinical diagnostic criteria which do not get to differentiate this disease accurately from other causes of dementia^1^.

Before dementia phase is established, cognition problems develop in a slow but progressive way, and can interfere limitedly in daily activities. This prodromal stage, called mild cognitive impairment (MCI), is a clinically heterogeneous syndrome and a consequence of different etiologies. Its definition has expanded in recent years^2–4^. MCI symptoms can also be stable for many years or even disappear; however, it is clear that the amnestic and multidomain MCI raises the progression risk to AD^5,6^. It is complicated to make a correct diagnosis of AD, especially in its MCI stage; therefore, the search for low-cost and innocuous biomarkers is important^7^. Even when a set of biomarkers have been approved and incorporated into the new clinical diagnostic criteria^8,9^, most demonstrate suboptimal test precision and involve either prohibitive costs or substantially invasive processes^10,11^.

The retina is a part of the central nervous system (CNS). with shared embryological origins. Unmyelinated axons of neurons in the ganglion cell layer (CGL) build the retinal nerve fiber layer (RNFL). These fibers continue as the optic nerve into the brain.

Optical coherence tomography (OCT) is a cheap, efficient and noninvasive transpupillary technique that facilitates objective *in vivo* retinal quantification^12^. OCT is utilized regularly in clinical ophthalmology to assess retinal integrity and is a promising tool for neurological investigation because of its high correlation with a number of visual electrophysiological tests^13,14^ and considerable reliability in a wide range of neurological pathologies^15,16^.

Visual symptoms such as impairment of both contrast and color sensitivity and perception of motion and depth are regularly observed in AD and other neurodegenerative conditions^17,18^. They are typically considered a consequence of damage to associative visual cortical areas^19,20^; however, there is growing evidence that neuroretinal involvement may also be a contributing factor^21^, and this has generated interest in the quest for retinal AD biomarkers^22,23^. A significant number of postmortem pathological studies have outlined RNFL and GCL reduction in AD patients^24,25^, although others have provided divergent results^26^.

Retinal thinning is found in AD and many other CNS conditions, including neuromyelitis optica, Parkinson’s disease^27,28^. One hypothesis highlights that it is a consequence of retrograde degeneration of the axons of the CGL or the typical pathological deposits of Alzheimer’s Disease in the retina. Indeed, initial histological studies^24,25^ have not identified neurofibrillary tangles or beta-amyloid plaques in AD patients’ retina. However more recent studies state to have found them^29,30^. The macula contains most of the retinal neurons’ bodies, and therefore, macular volume evaluation may determine neuronal loss, showing if and how the neurodegeneration is taking place. Controls appear to have a higher macular volume than AD but lower relative to amnestic MCI^31^. Gliosis or inflammation in the prodromal phase of AD might be a possible explanation for this finding.

Studies on macular thinning are not fully conclusive because of their small sample size, significant heterogeneity in their methods^32–34^, and divergent results. Compared with cognitively healthy persons, most studies provide evidence of macular volume and thickness reduction in people with cognitive impairment (either MCI or AD), affecting primarily either the inner and outer rings^25,35^ or the fovea^15^. With one exception^31^, people with AD show a larger reduction in macular thickness than those with MCI in articles cited earlier. Macular layer segmentation reveals CGL and RNFL atrophy in people with AD and is associated with disease severity^36^ and axonal damage. However, recent studies do not provide evidence of retinal thinning in the macula^37,38^ and the optic disc^39,40^.

The goal of this paper is to assess the clinical usefulness and viability of the analysis of all main macular parameters obtained through OCT automatic segmentation in the differentiation of controls, MCI, and AD in the work routine of a memory unit (MU).

## Results

**Figure 1:**
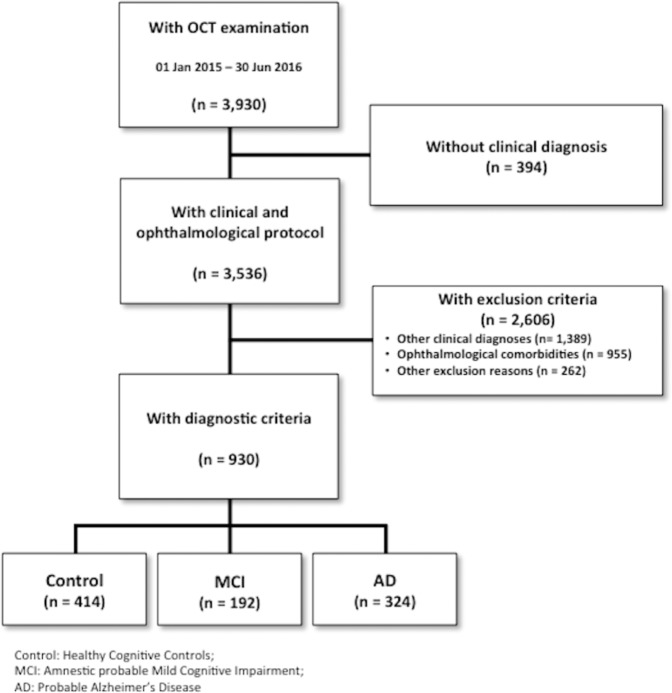
Patient selection and study cohort flowchart. Eligible population and selection of the study sample for this study through inclusion and exclusion criteria. Figure 1 was published previously in https://www.nature.com/articles/s41598-018-34577-3.pdf.

A total of 3,930 people attending a MU participated in this study. Ninety percent of them were given a clinical diagnosis. The selection algorithm’ s details is depicted in Fig. 1. An amount of 955 participants were excluded because of several eye diseases. Among them, the most prevalent^41^ were glaucoma (30,6%) and degenerative maculopathy (30,3%).

A total of 930 subjects were deemed to meet all the inclusion criteria and none of the exclusion criteria: 414 people were allocated to the control group, 192 to the MCI one, and 324 to the Alzheimer’s Disease group. Their demographical features are shown in Table 1. Cognitively healthy persons were the youngest and demonstrated the greatest educational attainment. They showed the best Mini-Mental State Examination (MMSE) scores as well. The MCI patients were younger and had better MMSE scores than the AD group.

**Table 1:** Baseline demographics.

		Mean	SD	Intergroup Significance
Education (years)	Control	10.96	4.13	<0.001^+^
	MCI	7.00	4.32	
	AD	6.14	4.08	
	Total	8.46	4.72	
Age (years)	Control	65.93	9.01	<0.001^+^
	MCI	76.46	7.14	
	AD	78.99	7.87	
	Total	73.05	10.23	
MMSE (points)	Control	29.29	1.00	<0.001^+^
	MCI	25.14	2.97	
	AD	20.28	3.98	
OCT Image Quality (%)	Control	47.81	7.57	<0.001^+^
	MCI	44.59	8.23	
	AD	43.23	10.33	
	Total	45.41	9.09	
Gender (% women)	Control	67.7%		<0.001^*^
	MCI	56.2%		
	AD	74.0%		
	**Total**	**67.8%**		

Demographic features including age, gender, education, MMSE scores, and OCT quality image among groups are summarized. All the analyzed characteristics were significantly different among diagnostic groups.

^*^Pearson’s chi^2^ test;

^+^1-factor ANOVA;

Table 1 was published previously in https://www.nature.com/articles/s41598-018-34577-3.pdf.

Each covariate’s contribution to mean retinal thickness variance is summarized in Table 2. The key factor explaining the variability of macular thickness was age, which demonstrated a greater effect size and correlation than the diagnosis itself. OCT image quality did not significantly affect macular thickness variability among diagnostic groups.

**Table 2:** Contribution of every covariate to variance of the macular thickness.

Covariate	Significance	D.f.	Partial Eta^2^
Education	0.859	1	0.000
Gender	0.053	1	0.004
Age	0.0001	1	0.070
OCT image quality	0.639	1	0.000
Diagnosis	0.564	2	0.001

Correlation between demographical and ophthalmic covariates with the dependent variable is shown.

D.f.: degrees of freedom.

**Figure 2:**
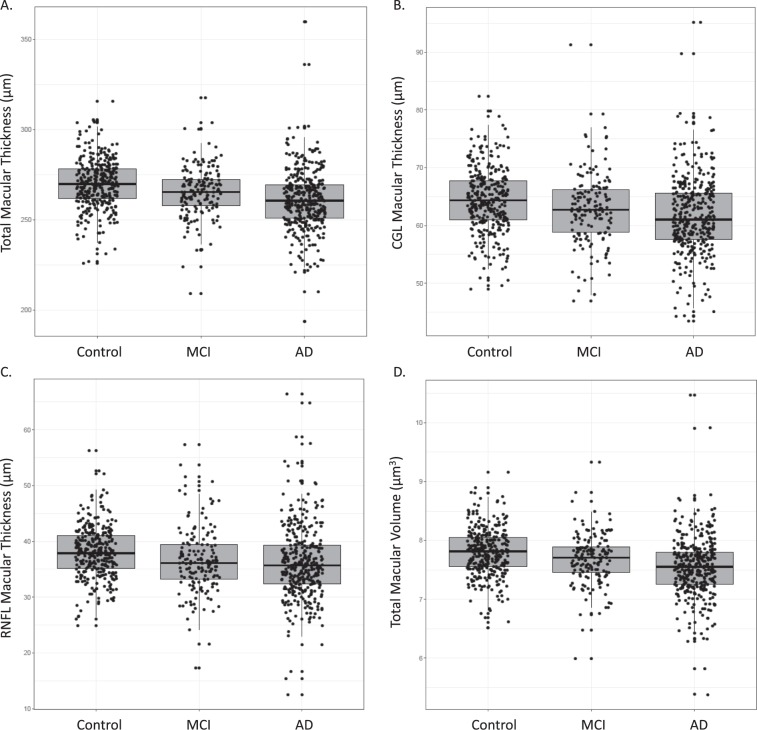
Macular parameters for each diagnostic group. This box plot represents the macular parameter for each diagnostic group (control, MCI, AD). No differences have been found among the three clinical categories. The bottom and top of the box are the first and third quartiles, and the band inside the box represents the median. AD: Alzheimer’s disease; MCI: mild cognitive impairment. (**A**) Macular Thickness (Total). (**B**) Macular Thickness (CGL). (**C**) Macular Thickness (RNFL). (**D**) Total Macular Volume.

We analyzed several macular parameters: mean thickness and volume (Table 3), thickness of macular layers (Table 4), and ETDRS sectors (Table 5). A box plot for macular variables was shown: total thickness (Fig. 2A), CGL thickness (Fig. 2B) RNFL thickness (Fig. 2C) and volume (Fig. 2D). Given the demographic differences among groups, we adjusted all macular variables in a multivariate model, including the next covariates: age, education, gender and OCT image quality. No significant differences in total (p = 0.564), CGL (p = 0.267), RNFL (p = 0.574), and macular thickness and volume (p = 0.380) were identified among diagnostic groups. Macular thickness was also compared regionally using Early Treatment Diabetic Retinopathy Study (ETDRS)–defined areas^42^. The superior (p = 0.040) and nasal (p = 0.040) sectors of the inner macular ring were the only macular regions showing significant differences among groups. The effect size was modest: the width of controls was only about 4 μm higher than the AD patients in both variables. However, significance disappeared after adjustment for multiple comparisons.

**Table 3:** Mean macular thickness and volume differences among diagnostic groups.

Group (N)	Mean	SD	Mean^aa^	SEM
Mean macular thickness p = 0.482				
Control (414)	275.28	13.95	271.52^aa^	0.87
MCI (192)	270.66	15.24	272.51^aa^	1.18
AD (324)	267.09	17.76	270.83^aa^	0.94
**Macular volume p** = **0.380**				
Control (414)	7.78	0.39	7.68^aa^	0.02
MCI (192)	7.65	0.43	7.70^aa^	0.03
AD (324)	7.54	0.51	7.65^aa^	0.03

Raw and adjusted mean overall total macular thickness (μm) and volume (μm^3^), standard deviation (SD), and standard error of the mean (SEM). After a multivariate adjustment (^aa^), no significant differences among diagnostic groups were detected. Dispersion data are shown as SEM.

SD: standard deviation; ^aa^after adjustment; SEM: standard error of the mean; p: significance; AD: Alzheimer’s disease; MCI: mild cognitive impairment.

**Table 4:** Mean macular layer differences among diagnostic groups.

Variable	Mean	SD	Mean^aa^	SEM
Ganglion cell layer width p = 0.267				
Control (414)	64.11	5.38	62.59^aa^	0.33
MCI (192)	62.68	6.27	63.51^aa^	0.42
AD (324)	61.58	6.55	63.05^aa^	0.36
**Mean macular RNFL width p** = **0.574**				
Control (414)	37.91	4.56	37.22^aa^	0.32
MCI (192)	36.30	6.01	36.67^aa^	0.41
AD (324)	36.11	6.62	36.79^aa^	0.35

Raw and adjusted mean overall total macular thickness (μm), standard deviation (SD), and standard error of the mean (SEM). After a multivariate adjustment (^aa^), no significant differences among diagnostic groups were detected. Dispersion data are shown as SEM.

SD: standard deviation; ^aa^after adjustment; SEM: standard error of the mean; p: significance; AD: Alzheimer’s disease; MCI: mild cognitive impairment.

**Table 5:** Macular sectors’ (ETDRS regions) thickness differences among diagnostic groups.

Group (N)	Mean	SD	Mean^aa^	SEM
ETDRS Center p = 0.735				
Control (414)	247.82	22.52	246.29^aa^	1.48
MCI (192)	246.76	25.59	246.89^aa^	1.91
AD (324)	243.19	30.03	245.08^aa^	1.61
**ETDRS Inner-Temporal p** = **0.125**				
Control (414)	299.92	16.03	296.04^aa^	1.10
MCI (192)	293.86	19.97	295.30^aa^	1.42
AD (324)	288.50	23.59	292.70^aa^	1.20
**ETDRS Inner-Superior p** = **0.040**				
Control (414)	312.45	16.22	307.81^aa^	1.12
MCI (192)	306.04	20.08	308.21^aa^	1.44
AD (324)	299.45	24.29	304.12^aa^	1.22
**ETDRS Inner-Nasal p** = **0.040**				
Control (414)	313.25	17.30	309.43^aa^	1.14
MCI (192)	306.00	21.21	307.53^aa^	1.47
AD (324)	300.75	23.35	304.74^aa^	1.24
**ETDRS Inner-Inferior p** = **0.095**				
Control (414)	309.88	16.22	305.44^aa^	1.12
MCI (192)	303.72	20.36	305,70^aa^	1.44
AD (324)	297.71	24.06	302.24^aa^	1.22
**ETDRS Outer-Temporal p** = **0.683**				
Control (414)	252.95	16.95	249.78^aa^	1.05
MCI (192)	248.50	18.13	249.81^aa^	1.35
AD (324)	245.24	20.85	248.54^aa^	1.14
**ETDRS Outer-Superior p** = **0.306**				
Control (414)	268.68	14.84	264.59^aa^	0.94
MCI (192)	264.31	17.20	266.51^aa^	1.24
AD (324)	260.31	20.01	264.25^aa^	1.05
**ETDRS Outer-Nasal p** = **0.397**				
Control (414)	285.41	17.711	281.18^aa^	1.04
MCI (192)	281.28	18.226	283.61^aa^	1.34
AD (324)	278.55	20.230	282.60^aa^	1.13
**ETDRS Outer-Inferior p** = **0.819**				
Control (414)	258.07	14.391	254.62^aa^	0.95
MCI (192)	253.72	16.979	255.57^aa^	1.23
AD (324)	251.43	19.813	254.78^aa^	1.04

Different raw and adjusted macular thickness (μm), standard deviation (SD), and standard error of the mean (SEM). After a multivariate adjustment, significant differences between diagnostic groups have appeared in the superior and nasal areas of the inner ring. After a correction for multiple comparisons, no significant differences among diagnostic groups were detected. Dispersion is shown as SEM.

## Discussion

We have conducted a cross-sectional study about differences in macular measurements in healthy controls and MCI and AD dementia groups, employing a large sample of varied ages, cognitive stages, and metabolic diseases to improve its ecological validity. The only requisite to allow participant with a particular disease into the study was that it did not alter retinal structure or cause OCT artifacts. For example, diabetic patients were permitted as long as they did not have diabetic retinopathy.

Our data show that the differences on macular quantifications between controls and AD patients are so small that such changes are less than both the intraindividual reliability of the technique and the age effect. (For example, mean macular thickness has less than 1 μm difference after adjustment.) Therefore, the use of macular thickness as a biomarker in a memory unit might not be sufficiently reliable so far.

Most patients were able to collaborate during OCT performance. The study’s sample size was one of the largest collected so far. Our study results contrasted with some previous works that found significant differences in macular thickness and volume between cognitive groups. Older literature on this matter had not provided solid conclusions so far. While some research suggested that macular thinning might correlate with cognitive diagnoses^25,35^, recent studies did not evidence any significant macular thickness reduction^37,38,43,44^, even using amyloid-proven status to confirm diagnoses^37^. One study even noted an increase in foveal thickness and volume in AD^38^. Our study showed a more prominent reduction in the macula’s inner ring than in the outer ring and not vice versa as was noted in a recent meta-analysis^32^. Pertinent to our findings may be the fact that the inner ETDRS zones contained more neurons; thus, neurodegeneration was expected to be most prominent there. The reduction did not demonstrate statistical significance adjusted by multiple comparisons. Contrary to other investigations^45^, our study did not identify any significant reduction in other macular layers such as RNFL or CGL.

The discrepancies observed in our data compared with previous literature could be related to several confounders. In the first instance, meta-analysis had evidenced heterogeneity in study design and in the clinical diagnosis of MCI and AD. Most previous studies were underpowered sample sizes and case-control designs where participants were prominently selected^32–34^. Only one eye per patient was included in some studies, while others included both. In addition, several OCT techniques and brands were used. Participant inclusion in most of former literature was not consecutive, and researchers were not blinded to the clinical diagnosis before OCT was performed, risking bias and overestimation of test accuracy^46,47^. Clinical criteria provided the basis for some studies, while others were supported by biomarkers. Furthermore, publication bias is identified, with an overrepresentation of smaller positive studies^32^, possibly leading to the true effect’s overestimation.

To avoid bias, first, we consecutively included every patient between 50 and 95 years of age who attended the MU no matter their cognitive picture and formal education. We used a standardized protocol for diagnosis that included extended neuropsychological test battery and neuroimaging procedures. The neurologist and optometrist were blinded to all actions executed on the same participant by their counterpart.

Second, probably given the difficulties inherent in recruiting cases and controls with extreme ages (old cognitive controls and young people with dementia), the assessed age range in existing studies was significantly limited (between 70 and 80 years in average). Cognitive impairment taking place at extreme ages was therefore not considered, although it was well-established that rates of cognitive decline varied according to the age^48^.

Third, AD and glaucoma were comorbid pathologies and shared mechanisms of pathophysiology with the same final result: retinal neurodegeneration^49^. It was therefore not easy to differentiate retinal changes due to AD from those caused by glaucoma. A similar dynamic was observed between AD and macular degeneration, another disease associated with age. Almost a quarter of eligible subjects in our cohort were not included because of ophthalmological comorbidities, primarily glaucoma and macular degeneration. Since these retinal pathologies also affected retinal thickness, the establishment of OCT as biomarker in a standard cognitive healthcare could be difficult or even unfeasible for a relatively large proportion of elderly subjects.

Factors that can significantly affect cognitive results, such as educational achievements or OCT image quality, were rarely considered in previous papers. Our study tested education, age, sex, and OCT image quality as covariates and demonstrated that age appeared to have the greatest influence in macular thickness variability. OCT signal strength was not an influential covariate in the macula, unlike in the optic disc^40^.

A few limitations of our study should be acknowledged. On one hand, our model’s covariates could not be enough to control intergroup variability due to potential eye confounders, such as axial length and optic disc area that have not been considered^50^. On the other hand, the study elicits only cross-sectional results, so we are not able to draw any substantive conclusions about how macular thinning can evolve. We are considering a longitudinal study to clarify the dynamics of macular thickness.

Many other OCT findings, including alterations in vascular layer and network, have been highlighted and could be relevant biomarkers for AD classification and progression^51–53^. In fact, successful retinal AD biomarkers might only be discovered after the integration of both neuroretinal and retinovascular in a composite biomarker. Advances in both OCT technology and inclusion of positron emission tomography (PET) and cerebrospinal fluid (CSF) biomarkers to ameliorate diagnostic certainty^54,55^ and to confirm the sensitivity and specificity of OCT could provide a greater insight into the relationship between brain pathology and retinal features.

## Methods

### Participant selection and characterization: the NORFACE cohort

The Neuro-Ophthalmology Research at Fundació ACE (NORFACE) cohort was established in 2014 to facilitate research in retinal biomarkers of AD and interrogate the thought-provoking relationships between retinal pathophysiology and AD. Recruitment of participants is prospective and consecutive from Fundació ACE-Institut Català de Neurociències Aplicades’s Memory Clinic in Barcelona, Spain. Standardized neuropsychological and medical examinations^56,57^ are used in a multidisciplinary approach to diagnose and care for patients with neurodegenerative diseases^58^.

As part of routine medical care and diagnostic workup, blood screening for syphilis, serum vitamin B12 and folate levels, and liver, renal, and thyroid functions are performed. Additionally, neuroimaging with structural brain MRI or head CT are employed to improve diagnostic certainty. These tests are followed by a clinical and neuropsychological examination and then reviewed by a multidisciplinary team to arrive at a consensus-based diagnosis^58^.

As part of the NORFACE cohort, each patient undergoes a complete neuro-ophthalmological interview and examination done by an optometrist. The assessment includes review of the ophthalmological history, visual acuity assessment, intraocular pressure measurement, and spectral domain OCT examination. The optometrist and neurologist have been blinded to each other’s evaluation data.

### Optical coherence tomography

Macular measurements were assessed by imaging patients with a 3D-OCT Maestro®, Fast Map software version 8.40 (Topcon Co., Tokyo, Japan). Importantly, the availability of high-resolution B-scan mode made pupil dilatation unnecessary. The OCT capture was merged with a real-color fundus picture captured by an internal camera.

The Topcon Advanced Boundary Segmentation (TABS) algorithm was employed to carry out automatic retina layer segmentation^59^. TABS has proven efficacious in treating ophthalmological diseases by providing accurate and consistent measurements of retina images across blood vessel shadows. Automatic segmentation seems to be less precise but more repeatable than manual one and this is an important issue in massive screening studies^60^. Macular thickness data were also shown in three concentric rings (ETDRS map) centered on the foveola. These were situated like this: a central macular ring, 1 mm from the fovea; an inner macular ring, 3 mm from the fovea; and an outer macular ring, 6 mm from the fovea. The inner and outer rings were each composed of four quadrants (superior, inferior, nasal, and temporal). Macular data were segmented in RNFL and CGL.

OCT data from only one eye (right) were analyzed for this study. The same optometrist screened all images for possible abnormalities after each OCT imaging session. Cases with abnormal findings were then reviewed by a consultant ophthalmologist who specialized in retinal pathology for a diagnostic report.

### Eligibility criteria

All individuals between 50 and 95 years of age consecutively evaluated at Fundació ACE´s Memory Clinic who fulfilled the control, MCI, or AD diagnostic criteria described below were considered eligible for inclusion in the study. The control group was selected for (a) the absence of significant symptoms (CDR = 0) and (b) having a normal age, gender, and education-adjusted performance on the Neuropsychological Battery of Fundació ACE (NBACE)^56,57^. The MCI group participants were required to (a) meet the Petersen criteria for amnestic MCI^61^ and (b) demonstrate an absence of significant signs of cerebrovascular or psychiatric disease. The last criterion was imposed to heighten the probability of AD as the underlying etiology for MCI^62^. The AD group was exclusively composed of subjects who met the National Institute of Neurologic and Communicative Disorders and Stroke-Alzheimer’s Disease and Related Disorders Association (NINCDS-ADRDA) criteria for probable AD^63^. Importantly, these inclusion criteria ensured the absence of other diseases capable of producing similar symptoms, thereby yielding a study cohort with high probability of “pure” AD etiology.

Patients were excluded from the study if they were unable to understand or collaborate in the neuro-ophthalmological evaluation, if there was only data derived from the left eye, or if there was the presence of OCT artifacts or diseases that might affect OCT measurement such as retinal or ocular diseases.

### Ethical considerations

The ethics committees of both the Hospital Clínic I Provincial and the Hospital Vall D’Hebron (Barcelona, Spain) approved this study and its observation of informed consent protocols in accordance with Spanish biomedical laws (Law 14/2007, July 3, about biomedical research; Royal Decree 1716/2011, November 18) and the guidelines set out in the Declaration of Helsinki. All participants signed the informed consent forms.

### Statistical analysis

Statistical analyses were carried out using IBM SPSS 20 (SPSS Inc., Chicago, IL) and in conformity with APOSTEL guidelines^64^. All data were tested for normality, skew, and restriction of range. All quantitative variables followed a normal distribution. The results of quantitative variables were presented as mean ± SD, while categorical variables were displayed by range, number and percentage.

The demographic attributes, clinical diagnoses, and OCT measurements were compared using the chi-squared test and parametric Student t-test. The differences in macular parameters between subgroups adjusted by age, education, gender, and image quality were tested using ANCOVA, with the different variables of macular thickness and volume as dependent factors and clinical groups as independent factors (three categories). Age, gender, years of education, and OCT image quality were factored into the model as adjustment variables. All the predictors’ explained variance was derived by calculating eta^2^ for each factor of the model. The threshold for a significant effect was set at p < 0.05.

## Data Availability

The data sets generated or analyzed during the current study are available from the corresponding author upon reasonable request.

## References

[1] Beach TG, Monsell SE, Phillips LE, Kukull W (2012). Accuracy of the Clinical Diagnosis of Alzheimer Disease at National Institute on Aging Alzheimer Disease Centers, 2005–2010. J. Neuropathol. Exp. Neurol..

[2] Morris JC (2001). Mild Cognitive Impairment Represents Early-Stage Alzheimer Disease. Arch. Neurol..

[3] Bondi MW (2014). Neuropsychological Criteria for Mild Cognitive Impairment Improves Diagnostic Precision, Biomarker Associations, and Progression Rates. J. Alzheimers. Dis..

[4] Petersen RC (2014). Mild cognitive impairment: A concept in evolution. J. Intern. Med..

[5] Espinosa A (2013). A longitudinal follow-up of 550 mild cognitive impairment patients: evidence for large conversion to dementia rates and detection of major risk factors involved. J. Alzheimers. Dis..

[6] Gainotti G, Quaranta D, Vita MG, Marra C (2014). Neuropsychological predictors of conversion from mild cognitive impairment to Alzheimer’s disease. J. Alzheimers. Dis..

[7] Jack CR, Holtzman DM (2013). Biomarker modeling of Alzheimer’s disease. Neuron.

[8] McKhann G (2011). The diagnosis of Dementia due to Alzheimer’s disease: Recommendations from the National Institute on Aging-Alzheimer’s Association workgroups on diagnostic guidelines for Alzheimer’s disease. Alzheimers. Dement..

[9] Dubois B (2010). Revising the definition of Alzheimer’s disease: A new lexicon. Lancet Neurol..

[10] Engler H (2006). Two-year follow-up of amyloid deposition in patients with Alzheimer’s disease. Brain.

[11] Diniz BSO, Pinto JA, Forlenza OV (2008). Do CSF total tau, phosphorylated tau, and β-amyloid 42 help to predict progression ofmild cognitive impairment to Alzheimer’s disease? A systematic review and meta-analysis of the literature. World J. Biol. Psychiatry.

[12] Puliafito CA (1995). Imaging of macular diseases with optical coherence tomography. Ophthalmol..

[13] Kromer R, Serbecic N, Hausner L, Froelich L, Beutelspacher SC (2013). Comparison of visual evoked potentials and retinal nerve fiber layer thickness in Alzheimer’s disease. Front. Neurol..

[14] Parisi V (2001). Correlation between optical coherence tomography, pattern electroretinogram, and visual evoked potentials in openangle glaucoma patients. Ophthalmol..

[15] Polo V (2014). Reliability and validity of Cirrus and Spectralis optical coherence tomography for detecting retinal atrophy in Alzheimer’s disease. Eye.

[16] Garcia-Martin E (2012). Ability and reproducibility of Fourier-domain optical coherence tomography to detect retinal nerve fiber layer atrophy in Parkinson’s disease. Ophthalmol..

[17] Iseri PK, Altinas Z, Tokay T, Yüksel N (2006). Relationship between Cognitive Impairment and Retinal Morphological and Visual Functional Abnormalities in Alzheimer Disease. J. Neuro-Ophthalmol..

[18] Katz B, Rimmer S (1989). Ophthalmologic manifestations of Alzheimer’s disease. Surv. Ophthalmol..

[19] Cogan DG (1985). Visual disturbances with focal progressive dementing disease. Am. J. Ophthalmol..

[20] Whitehouse PJ, Price DL, Clark AW, Coyle JT, DeLong MR (1981). Alzheimer disease: evidence for selective loss of cholinergic neurons in the nucleus basalis. Ann. Neurol..

[21] Berisha F, Feke GT, Trempe CL, McMeel JW, Schepens CL (2007). Retinal abnormalities in early Alzheimer’s disease. Invest. Ophthalmol. Vis. Sci..

[22] MacGillivray TJ (2014). Retinal imaging as a source of biomarkers for diagnosis, characterization and prognosis of chronic illness or long-term conditions. Br. J. Radiol..

[23] Ikram MK, Cheung CY, Wong TY, Chen CPLH (2012). Retinal pathology as biomarker for cognitive impairment and Alzheimer’s disease. J. Neurol. Neurosurg. Psychiatry.

[24] Hinton DR, Sadun AA, Blanks JC, Miller CA (1986). Optic- nerve degeneration in Alzheimer’s disease. N. Engl. J. Med..

[25] Sadun AA, Bassi CJ (1990). Optic nerve damage in Alzheimer’s disease. Ophthalmol..

[26] Davies DC, McCoubrie P, McDonald B, Jobst KA (1995). Myelinated axon number in the optic nerve is unaffected by Alzheimer’s disease. Br. J. Ophthalmol..

[27] Ratchford JN (2009). Optical coherence tomography helps differentiate neuromyelitis optica and MS optic neuropathies. Neurol..

[28] Martinez-Lapiscina EH (2016). Retinal thickness measured with optical coherence tomography and risk of disability worsening in multiple sclerosis: A cohort study. Lancet Neurol..

[29] Jentsch S (2015). Retinal fluorescence lifetime imaging ophthalmoscopy measures depend on the severity of Alzheimer’s disease. Acta Ophthalmol..

[30] Dentchev T, Milam AH, Lee VM, Trojanowski JQ, Dunaief JL (2003). Amyloid-beta is found in drusen from some age-related macular degeneration retinas, but not in drusen from normal retinas. Mol. Vis..

[31] Ascaso FJ (2014). Retinal alterations in mild cognitive impairment and Alzheimer’s disease: an optical coherence tomography study. J. Neurol..

[32] Den Haan J, Verbraak FD, Visser PJ, Bouwman FH (2017). Retinal thickness in Alzheimer’s disease: A systematic review and meta-analysis. Alzheimer’s Dement. Diagnosis..

[33] Coppola G (2015). Optical Coherence Tomography in Alzheimer’s Disease: A Meta-Analysis. PLoS One.

[34] Thomson KL, Yeo JM, Waddell B, Cameron JR, Pal S (2015). A systematic review and meta-analysis of retinal nerve fiber layer change in dementia, using optical coherence tomography. Alzheimers. Dement. Diagnosis, Assess. Dis. Monit..

[35] Larrosa JM (2014). Potential new diagnostic tool for Alzheimer’s disease using a linear discriminant function for Fourier domain optical coherence tomography. Invest. Ophthalmol. Vis. Sci..

[36] Garcia-Martin E (2016). Ganglion cell layer measurements correlate with disease severity in patients with Alzheimer’s disease. Acta Ophthalmol..

[37] Den Haan J (2019). Retinal Imaging Retinal thickness as a potential biomarker in patients with amyloid-proven early-and late-onset Alzheimer’s disease. Alzheimer’s Dement. Diagnosis.

[38] Poroy C, Yücel AÂ (2018). Optical Coherence Tomography: Is Really a New Biomarker for Alzheimer’s Disease?. Ann. Indian. Acad. Neurol..

[39] Kromer R (2014). Detection of Retinal Nerve Fiber Layer Defects in Alzheimer’s Disease Using SD-OCT. Front. psychiatry.

[40] Sánchez D (2018). Usefulness of peripapillary nerve fiber layer thickness assessed by optical coherence tomography as a biomarker for Alzheimer’s disease. Sci. Rep..

[41] Marquié M (2019). Visual impairment in aging and cognitive decline: experience in a Memory Clinic. Sci. Rep..

[42] Grading diabetic retinopathy from stereoscopic color fundus photographs–an extension of the modified Airlie House classification. ETDRS report number 10 (1991). Early Treatment Diabetic Retinopathy Study Research Group. Ophthalmol..

[43] Lad EM (2018). Evaluation of inner retinal layers as biomarkers in mild cognitive impairment to moderate Alzheimer’s disease. PLoS One.

[44] Knoll B (2016). Retinal nerve fiber layer thickness in amnestic mild cognitive impairment: Case-control study and meta-analysis. Alzheimers. Dement..

[45] Salobrar-Garcia E (2015). Analysis of Retinal Peripapillary Segmentation in Early Alzheimer’s Disease Patients. Biomed. Res. Int..

[46] Lijmer JG (1999). Empirical evidence of design-related bias in studies of diagnostic tests. J.A.M.A..

[47] Whiting P (2004). Sources of variation and bias in studies of diagnostic accuracy: a systematic review. Ann. Intern. Med..

[48] Bernick C, Cummings J, Raman R, Sun X, Aisen P (2012). Age and rate of cognitive decline in Alzheimer disease: implications forclinical trials. Arch. Neurol..

[49] Ghiso J, Doudevski I, Ritch R, Rostagno A (2013). Alzheimer’s disease and glaucoma: mechanistic similarities and differences. J. Glaucoma.

[50] Budenz DL (2007). Determinants of normal retinal nerve fiber layer thickness measured by Stratus OCT. Ophthalmol..

[51] Marziani E (2013). Evaluation of retinal nerve fiber layer and ganglion cell layer thickness in Alzheimer’s disease using spectral domain optical coherence tomography. Invest. Ophthalmol. Vis. Sci..

[52] Cheung CYL (2014). Microvascular network alterations in the retina of patients with Alzheimer’s disease. Alzheimers. Dement..

[53] de Jong FJ (2011). Retinal vascular caliber and risk of dementia: the Rotterdam study. Neurol..

[54] Ossenkoppele R (2015). Prevalence of amyloid PET positivity in dementia syndromes: a meta-analysis. J.A.M.A..

[55] Bouwman FH (2009). CSF biomarker levels in early and late onset Alzheimer’s disease. Neurobiol. Aging.

[56] Alegret M (2012). Normative data of a brief neuropsychological battery for Spanish individuals older than 49. J. Clin. Exp. Neuropsychol..

[57] Alegret M (2013). Cut-off scores of a Brief Neuropsychological Battery (NBACE) for Spanish Individual adults older than 44 years old. PLoS One.

[58] Boada M (2014). Design of a comprehensive Alzheimer’s disease clinic and research center in Spain to meet critical patient and family needs. Alzheimers. Dement..

[59] Yang Q (2010). Automated layer segmentation of macular OCT images using dual-scale gradient information. Opt. Express.

[60] Nagarkatti-Gude N (2019). Optical Coherence Tomography Segmentation Errors of the Retinal Nerve Fiber Layer Persist Over Time. J. Glaucoma..

[61] Petersen RC (2001). Practice parameter: early detection of dementia: mild cognitive impairment (an evidence-based review). Report of the Quality Standards Subcommittee of the American Academy of Neurology. Neurol..

[62] Lopez OL (2003). Prevalence and Classification of Mild Cognitive Impairment in the Cardiovascular Health Study Cognition Study. Arch. Neurol..

[63] McKhann G (1984). Clinical diagnosis of Alzheimer’s disease: report of the NINCDS-ADRDA Work Group under the auspices of Department of Health and Human Services Task Force on Alzheimer’s Disease. Neurol..

[64] Cruz-Herranz A (2016). The APOSTEL recommendations for reporting quantitative optical coherence tomography studies. Neurol..

